# Significant association between the Axin2 rs2240308 single nucleotide polymorphism and the incidence of prostate cancer

**DOI:** 10.3892/ol.2014.2177

**Published:** 2014-05-26

**Authors:** CHAO MA, CHUNXIAO LIU, PENG HUANG, HARUKI KAKU, JIE CHEN, KAI GUO, HIDEO UEKI, AKIKO SAKAI, YASUTOMO NASU, HIROMI KUMON, KENJI SHIMIZU, MASAMI WATANABE

**Affiliations:** 1 Department of Urology, Zhujiang Hospital, Southern Medical University, Guangzhou, Guangdong, P.R. China; 2 Center for Innovative Clinical Medicine, Okayama University Hospital, Okayama University, Graduate School of Medicine, Dentistry and Pharmaceutical Sciences, Okayama, Japan; 3 Department of Urology, Okayama University, Graduate School of Medicine, Dentistry and Pharmaceutical Sciences, Okayama, Japan; 4 Department of Molecular Genetics, Okayama University, Graduate School of Medicine, Dentistry and Pharmaceutical Sciences, Okayama, Japan

**Keywords:** prostate cancer, single nucleotide polymorphism, Axin2, rs2240308, Wnt signaling pathway

## Abstract

The Wnt signaling pathway plays a crucial role in human cancer development, and axis inhibition protein 2 (Axin2) is a master scaffold protein involved in Wnt signaling. Axin2 negatively regulates Wnt signaling and acts as a tumor suppressor protein. The present study evaluated the association between the Axin2 single nucleotide polymorphism (SNP) rs2240308 [guanine (G)/adenine (A)] and the incidence of prostate cancer. In total, 103 patients with prostate cancer and 100 cancer-free control males were included in this case-control study, and were genotyped using the genomic DNA extracted from peripheral blood samples. The results revealed a higher incidence of prostate cancer in the subjects with the homozygous GG genotype and a reduced cancer incidence in the patients with the GA genotype of the rs2240308 SNP (G/A) in the Axin2 gene. The adjusted odds ratio for carriers with the GA genotype was 0.377 (95% CI, 0.206–0.688; P=0.001) and that for the AA genotype was 0.830 (95% CI, 0.309–2.232; P=0.712) compared with the GG genotype. Therefore, the GA genotype was found to exhibit a protective effect that decreased the risk of prostate cancer. To the best of our knowledge, this is the first study to demonstrate the significant association between this SNP (rs2240308, G/A) and the risk of prostate cancer. This association indicates the possibility that the variations in the Axin2 gene in this position may play a significant role in promoting the development of cancer in the prostate. We believe that the Axin2 SNP (rs2240308) could be a useful biomarker for the predisposition and early diagnosis of the disease.

## Introduction

Prostate cancer is a common malignancy and one of the most frequent causes of mortality and morbidity in males in Western and Asian countries (1). Although the precise mechanism(s) underlying the development of prostate cancer are unknown, genetic factors, lifestyle and environmental factors are all involved (2). Genetically, prostate cancer develops as a result of mutational events involving the activation of proto-oncogenes and the inactivation of tumor suppressor genes (3). Numerous genetic variants have previously been found to be associated with an increased risk of prostate cancer in a genome-wide association study (4).

The Wnt signaling pathway plays a crucial role in human cancer development (5,6). Wnt signaling stabilizes cytoplasmic β-catenin, which stimulates the expression of cancer-related genes, including c-myc, c-jun and cyclin D1 (7,8). Axis inhibition protein 1 (Axin1) and its homolog, Axin2, are important components of the Wnt signaling pathway, and Axin2 negatively regulates Wnt signaling (9,10). Other molecules of the Wnt signaling pathway, including β-catenin and adenomatous polyposis coli (APC), interact with Axins, and the stability of β-catenin is regulated by the Axin complex (8). Therefore, Axin2 acts as a master scaffold protein in Wnt signaling, and is one of the tumor suppressor proteins.

The Axin2 gene consists of 10 exons encoding a 843-amino acid protein, and is located at human chromosome 17q24 (11,12). The mutation of the Axin2 gene and the loss of heterozygosity in the genomic locus have been observed in certain cancers, including colorectal, hepatocellular and ovarian cancer (13). It appears that the function of the Axin2 gene as a negative regulator of Wnt signaling is closely associated with the degradation of β-catenin, and in this way, affects carcinogenesis (9,10). Several types of single nucleotide polymorphisms (SNPs) have been reported in the Axin2 coding region, including rs2240308 (exon1), rs9915936 (exon5) and rs1133683 (exon5) (13). Among these Axin2 SNPs, a significant association between rs2240308 [guanine (G)/adenine (A); also described as 148 cytosine (C)/thymine (T), or CCT/TCT (Proline (Pro)/Serine (Ser)), or Pro50Ser] and the risk of lung cancer has been reported previously (14,15). It is therefore conceivable that the Axin2 rs2240308 SNP is involved in carcinogenesis and tumorigenesis in the prostate. However, the association between the SNP and prostate cancer has not been elucidated. In order to determine any association between the Axin2 polymorphism and the risk of prostate cancer, the present study focused on rs2240308 (G/A) and compared the polymorphic region between 103 patients with prostate cancer and 100 control males.

## Materials and methods

### Participants

Participants were recruited at Zhujiang Hospital (Guangzhou, Guangdong, China) between June 2012 and April 2013. The blood samples for the SNP analysis were obtained from the prostate cancer patients and matched control males during this period. All patients with prostate cancer were pathologically diagnosed using specimens from prostatic needle biopsies. The 103 patients with prostate cancer were classified as having either localized prostate cancer [T1-2, N0, M0; Gleason score 2–7; and prostate-specific antigen (PSA) levels of ≤50 ng/ml] or advanced prostate cancer (T3/4 or N+ or M+; or Gleason score 8–10; or PSA levels of >50 ng/ml). A histopathological diagnosis was made by an experienced pathologist, and the cancer was clinically staged according to the tumor-node-metastasis (TNM) classification (16). The histological grading of the biopsy specimens was performed using Gleason’s system by the same pathologist. The clinical TNM stage was used when the pathological TNM stage was unavailable, and the biopsy Gleason score was used when the pathological Gleason score of the prostatectomy specimens was unavailable (17,18).

The control subjects were volunteer cancer-free male patients or healthy males. All 100 controls were confirmed to be free from prostate cancer based on their PSA levels and digital rectal or ultrasound imaging examinations. The controls were selected during the recruitment period according to the expected age distribution and geographic origin of the case patients with prostate cancer. A questionnaire was also administered to the participants to determine their smoking status (never/ever, smoking duration in years and cigarettes/day), drinking status (never/ever, drinking history and grams of alcohol/day) and clinical information with regard to hypertension (without/with). This study was approved by the ethics committee of Zhujiang Hospital, and the purpose and requirements of the study were clearly explained to each participant. All patients provided written informed consent.

### DNA extraction and genetic analysis

A 2-ml blood sample was obtained from each participant, and kept at room temperature for no more than six hours. Genomic DNA was extracted using a TIANamp Blood DNA kit (Tiangen Biotech, Beijing, China) according to the manufacturer’s instructions, and was stored at −20°C. The Axin2 rs2240308 SNP and seven SNPs of other genes (data not shown) were analyzed using these samples. The genetic analyses were performed using the ABI SNaPshot multiplex system (Life Technologies, Carlsbad, CA, USA).

For the first PCR, multiple PCR amplification was carried out on a MyCycler Thermal Cycler (Bio-Rad Laboratories, Hercules, CA, USA) using 1 μl genomic DNA (10–50 ng) as a template, 2.5 μl 10× PCR buffer (Mg2+ free), 0.8 μl MgCI2 (50 mM), 0.5 μl dNTPs (2.5 mM each), 0.2 μl Platinum Taq (5 U/μl), 1 μl first primer mix and ddH_2_O to reach a final reaction volume of 25 μl. The program used was an initial denaturation step of 95°C for 5 min, followed by 33 cycles of 95°C for 30 sec, 56°C for 30 sec and 72°C for 30 sec. The final extension step was performed for 5 min at 72°C. The first PCR primers used for rs2240308 were: Forward, 5′-ACGTTGGATGGCTCCCCCAACCCATCTT-3′, and reverse, 5′-ACGTTGGATGGCGCTATGTTGGTGACT TGC-3′. The PCR products were confirmed on a 2% agarose gel. To avoid subsequent PCR-related reactions, the primers and unincorporated dNTPs of the preliminary PCR reaction were inactivated prior to the genetic analysis.

For the second PCR of the SNaPshot reaction, the PCR primer used for rs2240308 was 5′-TTTTTTTTTTTTTTTTTT TTTCTGGTGTTGGAAGAGACAG-3′. Since all the primers for eight SNP targets were used simultaneously, numerous T residues were attached to the typing primer of rs2240308 to discriminate it from the other targets. The single typing primer was used for typing a single SNP genotype with ddNTP end-labeling. SNaPshot reactions were carried out using 1 μl purified first PCR products as a template, with the reaction mix (1.5 μl) and probe mix (0.5 μl). Reactions were carried out in the MyCycler Thermal Cycler at 96°C for 10 sec, 51°C for 5 sec and 60°C for 30 sec, and were repeated for a total of 25 cycles. In an ABI optical plate (Life Technologies), 8.8 μl highly-deionized Formamide, 0.2 μl GeneScan 120 LIZ Size Standard and 1 μl of the reaction mixture were combined, and then were denatured at 95°C for 5 min. The samples were loaded on the ABI PRISM 3730 DNA Analyzer and analyzed using the GeneMapper (version 4.1) software program (both Life Technologies).

### Statistical analysis

The present study compared the proportion (percentage) of the each genotype and allele of the rs2240308 SNP (G/A) of the Axin2 gene in the controls and prostate cancer cases. The Axin2 SNP genotypes or alleles are shown as GG, GA and AA, or G and A, respectively. The association between the SNP and the incidence of prostate cancer was analyzed using a logistic regression model. The odds ratio (OR), 95% confidence interval (CI) and corresponding P-values for the association between the prostate cancer risk and the genotypes or alleles were calculated. The data for each genotype or allele was compared with that of the common homozygote or allele (GG or G, respectively) as the reference group. The analyses were also stratified by the age of the patient at diagnosis (≤72 or >72 years) and by the aggressiveness of the disease (localized or advanced prostate cancer, as aforementioned). In these analyses, the data were adjusted for the age, smoking status and drinking status, as described previously (14,19).

The data are shown as the mean ± standard deviation. The χ^2^ test was used to compare the distribution of the control males and prostate cancer patients or of the clinical characteristics. The Mann-Whitney U test was also performed to analyze the statistical significance of differences in the age and PSA level at diagnosis. All statistical analyses were conducted using the SPSS software program, version 17.0 (SPSS Inc., Tokyo, Japan). P<0.05 was considered to indicate a statistically significant difference.

## Results

The clinical features of the 103 patients with prostate cancer and 100 control males are shown in Table I. The mean age of the prostate cancer patients and the controls at the time that the blood was drawn was 71.2 and 70.4 years, respectively. There were no significant differences between the controls and cases with regard to age, smoking status, drinking status or the frequency of hypertension.

The proportions of each genotype and allele of the rs2240308 SNP (G/A) of the Axin2 gene in the controls and prostate cancer cases are shown in Table II. In the prostate cancer cases, the GG genotype was observed in a significantly higher proportion of patients (59.2%) compared with that in the controls (39.0%). By contrast, the GA genotype was observed in a significantly lower proportion of the cancer cases (30.1%) compared with the controls (52.0%). In the allele analysis, the G allele was observed more frequently in the cancer cases (74.3%) than in the controls (65.0%). The A allele was consistently observed more often in the controls (35.0%) than in the cancer cases (25.7%). The adjusted OR for the carriers with the GA genotype was 0.377 (95% CI, 0.206–0.688; P=0.001) and for those with the AA genotype was 0.830 (95% CI, 0.309–2.232; P=0.712) compared with the GG genotype. The difference between the G allele (as the reference) and the A allele between the cancer and control groups was also significant (adjusted OR, 0.583; 95% CI, 0.369–0.922; P=0.021). These data indicate that the incidence of prostate cancer in the patients with the GA genotype was significantly lower compared with that in the GG reference group, and that the GA genotype is therefore considered to be a factor that decreases the risk of prostate cancer.

In the subsequent stratified analyses, a diagnosis age of 72 years was chosen as the dividing line so that there would be similar numbers of younger and older participants. As shown in Table III, a significant association was found between the GA genotype and a reduced incidence of prostate cancer in the younger and older subgroups in comparison to the GG genotype as the reference. In the allele analysis, a significant association between the A allele and reduced prostate cancer incidence was only found in the younger subgroup (≤72 years), but was not observed in the older subgroup (>72 years).

When patients were stratification by the aggressiveness of the cancer (Table IV), the GA genotype was found to have a significant association with reduced prostate cancer development in the patients with localized and advanced disease in comparison to the GG reference genotype. For the A allele, a significant association with a lower incidence of prostate cancer was only found in the localized cancer subgroup, and was not observed in the advanced subgroup. Thus, the GA genotype is significantly associated with a lower incidence of prostate cancer, regardless of the age of patients and the aggressiveness of the cancer. By contrast, the A allele is associated with a reduced incidence of prostate cancer in younger patients and in patients with localized cancer.

Since the GG genotype indicated a tendency for a higher prostate cancer risk in terms of the rs2240308 SNP (G/A) of the Axin2 gene, the present study next analyzed the association between the GG or non-GG genotypes and the clinical characteristics of cancer aggressiveness, Gleason score, PSA level, age at diagnosis, smoking and drinking status and hypertension. Table V shows the results of the genotype distribution with regard to these clinical parameters. A positive association was observed between the GG risk genotype and the serum PSA level (ng/ml) at the time of diagnosis in the entire study group, indicating that a higher PSA level could be observed in the GG genotype subgroup in comparison to the non-GG subgroup. This finding is consistent with the results showing that the subgroup with the GG genotype contained more prostate cancer patients than the non-GG genotype subgroup. There was no significant association between the other clinical characteristics and the GG risk genotype, indicating that they had no interaction with the GG genotype.

## Discussion

The present study investigated the association between the rs2240308 SNP (G/A) of the Axin2 gene, a regulator of Wnt signaling, and the incidence of prostate cancer. The results revealed a higher risk of prostate cancer in the patients with the homozygous GG genotype and a reduced incidence of cancer in the patients with the GA genotype. Therefore, the GA genotype was found to be a protective genotype that decreased the risk of prostate cancer. To the best of our knowledge, this is the first study to demonstrate the significant association of the rs2240308 SNP (G/A) with the risk of prostate cancer. This association indicates the possibility that the variance in the Axin2 gene in this position may play a significant role in promoting the development of prostate cancer.

The Axin2 protein is a negative regulator of the Wnt signaling pathway due to its role in the degradation of β-catenin (9,10). Alterations in the Axin2 gene have been detected in several cancer types (13). Certain mutations of the Axin gene are found in the functional domains, including the β-catenin and APC binding sites, and sequence variants have been reported in colon and ovarian cancer (20). It has been reported that these mutations alter the interaction or binding of the Axin protein with the other Wnt signaling core proteins, including glycogen synthase kinase 3 (GSK3), frequently rearranged in advanced T-cell lymphomas 1 and disheveled (20,21), indicating that Axin2 dysfunction may be involved in carcinogenesis and tumor development.

In addition to the current association between the Axin2 rs2240308 SNP and prostate cancer in the Chinese population, other studies have demonstrated a significant association between the rs2240308 SNP and lung cancer development in Japanese (14) and Turkish (15) populations. Although it is not yet known how the rs2240308 SNP is associated with cancer, one mechanism may be explained by the alteration of the coding amino acid at the affected position of the Axin2 protein. In the Axin2 rs2240308 SNP, the change of the G allele to the A allele results in an amino acid alteration from Pro to Ser at codon 50. On the other hand, there have been several studies regarding the functional domains of the Axin2 protein as a component of Wnt signaling (9,14,22). The predicted functional domains of the Axin2 protein include the regulator of G protein signaling (RGS) domain (amino acids 81–200), the GSK3 interaction domain (amino acids 327–413) and the β-catenin binding site (amino acids 413–476) (14). It has been reported that the RGS domain works as the APC-binding site (9), and the location of the Axin2 rs2240308 SNP at codon 50 is extremely close to the RGS domain (14). Therefore, it is conceivable that the alternation of the interaction between Axin2 and APC may affect β-catenin regulation and permit carcinogenesis.

In conclusion, the present study demonstrates that there is a significant association between the rs2240308 SNP (G/A) of the Axin2 gene and the risk of prostate cancer. To the best of our knowledge, this is the first study showing the possible involvement of the Axin2 polymorphism in prostate cancer development. Although additional studies with larger and more diverse populations and a functional analysis of the polymorphism are necessary to confirm and extend our findings, we believe that the Axin2 rs2240308 SNP could be a useful biomarker for the predisposition to prostate cancer and for the early diagnosis of the disease.

## Figures and Tables

**Table I:** Clinical characteristics of the controls and patients.

Variables	Controls	Patients	Localized PCa	Advanced PCa	P-value^a^
No. of subjects	100	103	35	68	
Mean age, years (SD)	70.4 (10.9)	71.2 (8.9)	73.2 (7.7)	70.1 (9.4)	0.503
Mean PSA, (SD)	6.9 (8.0)	59.9 (94.1)	12.1 (11.5)	87.4 (109.0)	<0.001
Smoking status, n (%)					0.405
Never	80 (80.0)	87 (84.5)	33 (94.3)	54 (79.4)	
Ever	20 (20.0)	16 (15.5)	2 (5.7)	14 (20.6)	
Drinking status, n (%)					0.751
Never	91 (91.0)	95 (92.2)	33 (94.3)	62 (91.2)	
Ever	9 (9.0)	8 (7.8)	2 (5.7)	6 (8.8)	
Hypertension, n (%)					0.854
No	73 (73.0)	74 (71.8)	22 (62.9)	52 (76.5)	
Yes	27 (27.0)	29 (28.2)	13 (37.1)	16 (23.5)	
Gleason score, n (%)^b^					
Total	-	85 (100.0)	29 (100.0)	56 (100.0)	
2–6	-	23 (27.1)	20 (69.0)	3 (5.4)	
7	-	20 (23.5)	9 (31.0)	11 (19.6)	
8–10	-	42 (49.4)	0 (0.0)	42 (75.0)	
Tumor stage, n (%)^b^					
Total	-	97 (100.0)	31 (100.0)	66 (100.0)	
T1	-	5 (5.2)	5 (16.1)	0 (0.0)	
T2	-	42 (43.3)	26 (83.9)	16 (24.2)	
T3	-	32 (33.0)	0 (0.0)	32 (48.5)	
T4	-	18 (18.5)	0 (0.0)	18 (27.3)	
Nodal stage, n (%)					
Total	-	89 (100.0)	31 (100.0)	58 (100.0)	
N0	-	57 (64.0)	31 (100.0)	26 (44.8)	
N1	-	32 (36.0)	0 (0.0)	32 (55.2)	
Metastasis stage, n (%)					
Total	-	96 (100.0)	31 (100.0)	65 (100.0)	
M0	-	65 (67.7)	31 (100.0)	34 (52.3)	
M1	-	31 (32.3)	0 (0)	31 (47.7)	

a Analyzed between controls and all patients;

b Data was not available for all 103 participants.

**Table II:** Association of the Axin2 SNP genotype (GG, GA or AA) and allele (G or A) with the incidence of prostate cancer in the patients (n=103) and controls (n=100).

SNP	Genotype or allele	Controls, n (%)	Patients, n (%)	Crude OR (95% CI)	P-value	Adjusted OR^a^ (95% CI)	P-value
rs2240308							
	GG	39 (39.0)	61 (59.2)	1.000 (Reference)		1.000 (Reference)	
	GA	52 (52.0)	31 (30.1)	0.381 (0.209–0.694)	0.002	0.377 (0.206–0.688)	0.001
	AA	9 (9.0)	11 (10.7)	0.781 (0.297–2.058)	0.618	0.830 (0.309–2.232)	0.712
	non-GG	61 (61.0)	42 (40.8)	0.440 (0.251–0.772)	0.004	0.440 (0.250–0.774)	0.004
	G	130 (65.0)	153 (74.3)	1.000 (Reference)		1.000 (Reference)	
	A	70 (35.0)	53 (25.7)	0.643 (0.420–0.990)	0.042	0.583 (0.369–0.922)	0.021

a Adjusted for age, smoking status and drinking status in a logistic regression model.

SNP, single nucleotide polymorphism; OR, odds ratio; CI, confidence interval.

**Table III:** Frequencies of Axin2 SNP genotypes (GG, GA or AA) and alleles (G or A) and the adjusted OR stratified by the age at diagnosis, for patients (n=52) and controls (n=54) ≤72 years old, and for patients (n=51) and controls (n=46) >72 years old.

	Age at diagnosis: ≤72 years				Age at diagnosis: >72 years			
		
Genotype or allele	Controls, n (%)	Patients, n (%)	Adjusted OR^a^ (95% CI)	P-value	Controls, n (%)	Patients, n (%)	Adjusted OR^a^ (95% CI)	P-value
GG	21 (38.9)	32 (61.5)	1.00 (Reference)		18 (39.1)	29 (56.9)	1.00 (Reference)	
GA	26 (48.1)	16 (30.8)	0.42 (0.18–0.97)	0.042	26 (56.5)	15 (29.4)	0.36 (0.15–0.88)	0.025
AA	7 (13.0)	4 (7.7)	0.37 (0.09–1.46)	0.155	2 (4.4)	7 (13.7)	2.55 (0.45–14.27)	0.288
non-GG	33 (61.1)	20 (38.5)	0.41 (0.19–0.90)	0.025	28 (60.9)	22 (43.1)	0.51 (0.22–1.15)	0.104
G	68 (63.0)	80 (76.9)	1.00 (Reference)		62 (67.4)	73 (71.6)	1.00 (Reference)	
A	40 (37.0)	24 (23.1)	0.42 (0.22–0.81)	0.009	30 (32.6)	29 (28.4)	0.82 (0.43–1.59)	0.560

a Adjusted for age, smoking status and drinking status in a logistic regression model.

SNP, single nucleotide polymorphism; OR, odds ratio; CI, confidence interval.

**Table IV:** Frequencies of Axin2 SNP genotypes (GG, GA or AA) and alleles (G or A) and the adjusted OR stratified by the aggressiveness of the cancer, for controls (n=100) versus patients (n=35) with localized PCa, and for controls (n=100) versus patients with advanced PCa (n=68).

Genotype or allele	Controls, n (%)	Localized PCa, n (%)	Adjusted OR^a^ (95% CI)	P-value	Controls, n (%)	Advanced PCa, n (%)	Adjusted OR^a^ (95% CI)	P-value
GG	39 (39.0)	24 (68.6)	1.00 (Reference)		39 (39.0)	37 (54.4)	1.00 (Reference)	
GA	52 (52.0)	9 (25.7)	0.25 (0.10–0.61)	0.002	52 (52.0)	22 (32.4)	0.45 (0.23–0.87)	0.018
AA	9 (9.0)	2 (5.7)	0.38 (0.07–1.98)	0.249	9 (9.0)	9 (13.2)	1.06 (0.37–3.02)	0.921
non-GG	61 (61.0)	11 (31.4)	0.26 (0.11–0.62)	0.002	61 (61.0)	31 (45.6)	0.53 (0.28–0.99)	0.048
G	130 (65.0)	57 (81.4)	1.00 (Reference)		130 (65.0)	96 (70.6)	1.00 (Reference)	
A	70 (35.0)	13 (18.6)	0.41 (0.21–0.81)	0.010	70 (35.0)	40 (29.4)	0.67 (0.39–1.13)	0.133

a Adjusted for age, smoking status and drinking status in a logistic regression model.

SNP, single nucleotide polymorphism; OR, odds ratio; CI, confidence interval; PCa, prostate cancer.

**Table V:** Association of the Axin2 SNP genotypes (GG or non-GG) with the clinical characteristics.

Genotype	Cancer aggressiveness, n (%)		Gleason score, n (%)			PSA level at diagnosis	Age in years at diagnosis	Smoking status, n (%)		Drinking status, n (%)		Hypertension, n (%)	
				
	Localized	Advanced	2–6	7	8–10			Never	Ever	Never	Ever	No	Yes
Total n	35	68	23	20	42	189	203	167	36	186	17	56	147
GG	24 (68.6)	37 (54.4)	15 (65.2)	13 (65.0)	21 (50.0)	48.3 (n=91)	71.1 (n=100)	83 (49.7)	17 (47.2)	91 (48.9)	9 (52.9)	23 (41.1)	77 (52.4)
non-GG	11 (31.4)	31 (45.6)	8 (34.8)	7 (35.0)	21 (50.0)	18.6 (n=98)	70.5 (n=103)	84 (50.3)	19 (52.8)	95 (51.1)	8 (47.1)	33 (58.9)	70 (47.6)
P-value	0.166		0.370			<0.001	0.647	0.787		0.751		0.150	

SNP, single nucleotide polymorphism; PSA, prostate-specific antigen.
